# T4 bacteriophage as a phage display platform

**DOI:** 10.1007/s00203-014-0989-8

**Published:** 2014-05-15

**Authors:** Mariam Gamkrelidze, Krystyna Dąbrowska

**Affiliations:** 1 Agricultural University of Georgia, David Agmashenebeli Alley, 13th km, 0131 Tbilisi, Georgia; 2 Institute of Immunology and Experimental Therapy, Polish Academy of Sciences, ul. R. Weigla 12, 53-114 Wroclaw, Poland

**Keywords:** Bacteriophages, Phage display, Vaccine design, T4 phage, Hoc protein, Soc protein

## Abstract

Analysis of molecular events in T4-infected *Escherichia coli* has revealed some of the most important principles of biology, including relationships between structures of genes and their products, virus-induced acquisition of metabolic function, and morphogenesis of complex structures through sequential gene product interaction rather than sequential gene activation. T4 bacteriophages and related strains were applied in the first formulations of many fundamental biological concepts. These include the unambiguous recognition of nucleic acids as the genetic material, the definition of the gene by fine-structure mutation, recombinational and functional analyses, the demonstration that the genetic code is triplet, the discovery of mRNA, the importance of recombination and DNA replications, light-dependent and light-independent DNA repair mechanisms, restriction and modification of DNA, self-splicing of intron/exon arrangement in prokaryotes, translation bypassing and others. Bacteriophage T4 possesses unique features that make it a good tool for a multicomponent vaccine platform. Hoc/Soc-fused antigens can be assembled on the T4 capsid in vitro and in vivo. T4-based phage display combined with affinity chromatography can be applied as a new method for bacteriophage purification. The T4 phage display system can also be used as an attractive approach for cancer therapy. The data show the efficient display of both single and multiple HIV antigens on the phage T4 capsid and offer insights for designing novel particulate HIV or other vaccines that have not been demonstrated by other vector systems.

## Introduction

T-even bacteriophages are lytic bacterial viruses that infect *Escherichia coli*. These tailed DNA viruses share similar morphology and biochemical characteristics. They have been a major model system in the development of modern genetics and molecular biology since the 1940s; many investigations have taken advantage of their useful degree of complexity and the ability to deliver detailed genetic information by relatively simple experiments (Kutter et al. 1995; Miller et al. 2003). According to Miller et al. (2003), T4 bacteriophages and related strains were applied in the first formulations of many fundamental biological concepts. These include the unambiguous recognition of nucleic acids as the genetic material, the definition of the gene by fine-structure mutation, recombinational and functional analyses, the demonstration that the genetic code is triplet, the discovery of mRNA, the importance of recombination and DNA replications, light-dependent and light-independent DNA repair mechanisms, restriction and modification of DNA, self-splicing of intron/exon arrangement in prokaryotes, translation bypassing and others (Kutter and Wiberg 1969; Kutter et al. 1995; Miller et al. 2003). Analysis of molecular events in T4-infected *Escherichia coli* has revealed some of the most important principles of biology, including relationships between structures of genes and their products, virus-induced acquisition of metabolic function, and morphogenesis of complex structures through sequential gene product interaction rather than sequential gene activation (Karam 1994).

Posttranscriptional controls by T4 provide excellent systems for the study of RNA-dependent processes, particularly at the structural level. The redundancy of DNA replication and recombination systems of T4 reveals how phage and other genomes are stably replicated and repaired in various environments, providing insight into genome evolution and adaptations to new hosts and growth environments. Genomic sequence analysis in T4 has also provided new insights into bacteriophage molecular biology: tail fiber variations, lysis, gene duplications, and membrane localization of proteins, while high-resolution structural determination of the ‘cell-puncturing device,’ combined with the three-dimensional image reconstruction of the baseplate, has revealed the mechanism of penetration during infection. These relatively simple agents have been very important in the development of our understanding of all types of viruses, including human viruses, which are much more difficult to propagate and study. Despite that success, nearly 130 potential T4 phage genes remain uncharacterized. Similarities and differences among members of the T4 family are now being discovered, including those that infect bacteria other than *E. coli*. T4 functional genomics aids in the interpretation of these newly sequenced T4-related genomes and in broadening our understanding of the complex evolution and ecology of phages—the most abundant and among the most ancient biological entities on the Earth (Kutter et al. 1995; Miller et al. 2003).

## T4 phage capsid and nucleic acid

Bacteriophage T4 is a member of the *Myoviridae* family of the order *Caudovirales*. Bacteriophage T4 is a large, tailed, double-stranded DNA (dsDNA) virus. This phage has an exclusively lytic lifecycle (Kutter et al. 1995; Karam 1994; Baschong et al. 1991). The mature T4 virion, which contains 172-kbp genomic DNA and approximately 50 types of proteins, consists of a prolate head and a tail with a contractile sheath terminating in a base plate to which six long tail fibers are attached. The bacteriophage T4 head is an elongated icosahedron, 120 nm long and 86 nm wide, and is built with three essential proteins: gp23, which forms the hexagonal capsid lattice; gp24, which forms pentamers at eleven of the twelve vertices; and gp20, which forms the unique dodecameric portal vertex through which DNA enters during packaging and exits during infection (Rao and Black 2010; Fokine et al. 2004). The surface of the bacteriophage T4 capsid is coated with two decorative proteins, Hoc (highly antigenic outer capsid protein; molecular mass, 40 kDa) and Soc (small outer capsid protein; molecular mass, 9 kDa), at symmetrical positions on the icosahedron (160 copies of Hoc and 960 copies of Soc per capsid particle). Both these proteins are nonessential, i.e., not necessary for phage infectivity and viability. They are incorporated into the capsid surface after completion of capsid assembly (Karam 1994; Iwasaki et al. 2000).

The T4 long tail fibers are built from four different proteins, gene products: gp34, gp35, gp36, and gp37 (King and Laemmli 1971). These proteins form proximal and distal half-fiber segments of approximately 70 nm (Cerritelli et al. 1996), hinged at an angle of around 160°. Proximal half-fibers are composed of trimers of gp34, followed by a monomer of gp35 forming the hinge or ‘kneecap,’ whereas the distal half-fibers contain a trimer of gp36 and the most distal trimer of gp37. Gp 34 and gp37, as well as the short tail fiber protein gp12, require the chaperone gp57 for proper trimeric assembly; gp37 also requires gp38. Host recognition occurs through a reversible interaction of the tip of the long tail fibers with lipopolysaccharides or with the outer membrane porin proteins (Fokine et al. 2004, Bartual et al. 2010). In the area of the phage collar, fibritin (gp wac) is located as the phage collar whiskers. They form the six fibers that radiate from the phage neck; during phage morphogenesis, these whiskers bind the long tail fibers and facilitate their attachment to the phage baseplate (Letarov et al. 2005).

The past 20 years of research has greatly advanced the understanding of phage T4 head assembly and DNA packaging. Siyang et al. (2007) reported the direct measurements of single DNA molecule packaging in T4. They developed an optical tweezers method for measuring single DNA packaging dynamics in phage T4. A defined complex consisting of only proheads and gp17 ATPase can package DNA very rapidly, reconciling the ability of the virus to package its large genome in a limited time window during the natural infection process. Packaging can be initiated via a pathway in which prohead–gp17 complexes are formed and then rapidly bind and translocate DNA. The T4 motor can generate very high forces (>60 pN), suggesting that high force generation is a common property of viral motors. It can also translocate DNA at variable rates and reversibly pause and slip, capabilities that T4 and other viruses may need in order to regulate packaging with transcription, recombination, and repair processes. The development of this single-molecule assay, combined with recent determination of the crystal structure of the gp17–ATPase domain (Siyang et al. 2007; Sabanayagam et al. 2007), identification of critical functional residues of the motor and mutants, and recent development of a complementary fluorescence-correlation spectroscopy assay make T4 an attractive model system for detailed structure and function investigations (Fuller et al. 2007). The T4 head assembly proceeds via a number of intermediate stages: The genomic DNA is packaged into the prohead in a process that requires ATP energy (Black et al. 1994), a defined in vitro T4 packaging system consisting of only three components [empty proheads, the large terminase protein (gp17, the packaging ATPase), and DNA]. In the presence of ATP and under carefully optimized reaction conditions in bulk assays, as much as 50–100 % of the input DNA has been packaged (Black and Peng 2006; Sabanayagam et al. 2007).

## Phage display on the T4 particles

Phage display is an exponentially growing research area. It has had a major impact on immunology, cell biology, drug discovery, and pharmacology and is increasingly gaining importance in plant science (Willats 2002). In this technique, bacteriophage capsids are modified with foreign peptides or proteins. Specific DNA fragments encoding proteins can be inserted into genes encoding the phage capsid proteins; then, fusion proteins are produced and incorporated into the phage particle during assembly (Uchiyama et al. 2005). Phage display is an extremely powerful tool for selecting peptides or proteins with specific binding properties from vast amounts of variants. Put at its most simple, phage display is the presentation of a large number of foreign (nonphage) peptides, proteins, or antibody fragments at the surface of phage particles (Smith 1985; Winter et al. 1994; Kay and Hoess 1996; Hoess 2002).

As a system for the high throughput analysis of protein interactions, phage display is complementary to other methods such as yeast hybrid systems (Dress 1999), and each has its advantages and limitations. The most important advantage of phage display is the enormous diversity of variant proteins that can be represented. Phage display selection may be performed in vivo or in vitro (Johns et al. 2000). An attractive aspect of phage display is the possibility of constructing libraries in a simple, cheap, rapid way with no special equipment requirements (Willats 2002). A ‘phage display library’ is a heterogeneous mixture of such phage clones, each carrying a different foreign DNA insert and therefore displaying a different peptide on its surface. Each peptide in the library can replicate, since each displaying phage infects a bacterial host, multiplies, and produces a huge number of identical progeny phages displaying the same peptide (Smith and Petrenko 1997; Petrenko and Vodyanoy 2003).

Phage display usually applies filamentous phage strains: M13, fd, and f1. However, display systems based on bacteriophage T4 have also been developed, and they also seem to be very promising. The first approach to T4-based phage display was based on finding that minor T4 fibrous protein fibritin (gpwac) could be lengthened at the C terminus without impairing its folding or binding to the phage particle. The lengthened fibritin gene could easily be transferred into the T4 genome by homologous recombination with a plasmid containing the modified gene *wac*. The modified gene *wac* is expressed properly during phage reproduction, and the lengthened fibritin is assembled to phage particles. As an example of this type of method, the chimeric T4 particles were constructed. They carried a polypeptide of 53 residues, 45 of which were from the pre-S2 region of hepatitis B virus (Efimov et al. 1995).

Another possibility to use T4 capsid in a display system involves fusions to the decorative protein Soc or Hoc with foreign elements. This can be done both in vivo (in a bacterial cell, when the phage is being assembled) or in vitro, by adding the fusion to the defective capsid after phage propagation, or the *soc* or *hoc* gene can be flanked by specific T4 gene fragments to allow for homologous recombination with the T4 genome (Ren and Black 1998; Bratkovic 2010; Oślizło et al. 2011).

In vivo phage display technique employing natural assemblage in bacteria during a lytic growth cycle was applied for introducing fusion proteins to the phage capsid. The host bacteria expressed the fusion proteins from a designed expression vector. The T4 phage strains used in the experiments with supplementary expression vectors preferentially had a deletion or nonsense mutation of the *soc* or *hoc* gene. Since Hoc and Soc are not essential head proteins, these defects do not affect phage viability (Ren and Black 1998). Optimum localization of affinity tags presented on the phage capsid was determined by comparison of N- and C-termini in two decorative proteins of the T4 phage capsid: Hoc and Soc. The best position for effective presentation on the phage was the N-terminus of Hoc (Ceglarek et al. 2013).

The group of Venigalla Rao (Jiang et al. 1997) developed a phage display system, which allowed in-frame fusions of foreign DNA at a unique cloning site in the 5′ end of *hoc* or *soc.* A DNA fragment corresponding to the 36-amino acid PorA peptide from *Neisseria meningitidis* was cloned into the display vectors to generate fusions at the N-terminus of Hoc or Soc. The PorA-Hoc and PorA-Soc fusion proteins retained the ability to bind the capsid surface, and the foreign peptide was displayed in an accessible form as shown by its reactivity with specific monoclonal antibodies in an enzyme-linked immunosorbent assay. By employing T4 genetic strategies, they showed that more than one subtype-specific PorA peptide could be displayed on the capsid surface and that the peptide could also be displayed on a DNA-free empty capsid. The above properties of Hoc and Soc are uniquely suited to engineer the T4 capsid surface by arraying pathogen antigens. Ren et al. (1996) and Jiang et al. (1997) developed recombinant vectors that allowed fusion of pathogen antigens to the N- or C- termini of Soc and Hoc.

An in vitro display system has been developed taking advantage of the high affinity interactions between Hoc or Soc and the capsid (Shivachandra et al. 2006). In this system, antigens fused to Soc and Hoc with a hexa-histidine tag were overexpressed in *E. coli* and purified. The purified protein was assembled on Hoc^−^ Soc^−^ phage by simply mixing the purified components (Rao and Black 2010).

Hoc- or Soc-based phage display was also used in combination with affinity chromatography to provide a new method for phage purification. In this method, the T4 bacteriophage surface was furnished with standard affinity tags (GST, His-tag) that allowed phage binding to standard affinity resins. Affinity tags can be successfully incorporated into the T4 phage capsid by in vivo phage display, and they strongly elevate bacteriophage affinity to a specific resin (Oślizło et al. 2011; Ceglarek et al. 2013).

Permanent introduction of extraneous DNA into a phage genome is strongly unfavorable for medical purposes, mostly because of formal limitations to genetically modified organisms. Therefore, ‘competitive phage display’ was proposed as a variation in this method, instead of integration of foreign motifs with the phage genome. The phage was propagated in bacteria-expressing confluence of the phage protein Hoc with affinity tags from bacterial plasmids, independently from the phage expression system. Bacterial cells infected with a phage produce both wild types of the proteins and the protein fusions with affinity tags that were competitively incorporated into the phage head. This method allows purification of genetically native phages (Ceglarek et al. 2013).

Both Soc and Hoc proteins act as anchor proteins in the in vitro T4 display system. Recently, display of a macromolecular complex on T4 was reported: Two bipartite fusion proteins were constructed, the anthrax lethal factor (LF) fused to Hoc, and the N-terminal domain of the LF (LFn) coupled to Soc. Li et al. (2006) reported the first description of a macromolecular complex display system using bacteriophage T4, decorated with two dispensable outer capsid proteins: Hoc and Soc. Using a defined in vitro binding system, sequential assembly was performed by first attaching LF-Hoc and/or LFn-Soc to Hoc–Soc-phage, saturating the Hoc and Soc binding sites. Trypsin-nicked PA63 h (protective antigen heptamer) was then assembled into heptamers through specific interaction with the capsid-exposed LFn (Lethal Factor) domain. EF (Edema Factor) was then attached to the unoccupied sites of PA63 heptamers, completing the assembly of the tripartite anthrax toxin. Electron microscopy showed decoration of each capsid with a layer of heptameric PA63 rings. Up to 229 anthrax toxin complexes, equivalent to a total of 2,400 protein molecules and a mass of about 133 MDa (2.7 times the mass of the capsid shell), were anchored on a single particle, making it the highest density display reported on any virus. This work showed that the T4 capsid lattice provides a stable platform allowing maximum display of large hetero-oligomeric complexes in vitro and offers insights for developing novel vaccines, analysis of protein–protein interactions, and structure determination of complexes (Li et al. 2006).

## Applications of T4 phage-based display in vaccine design

One of the main applications of the T4 displayed antigen particles is their potential use in vaccine delivery. A number of independent studies have shown that the T4—displayed antigens without any added adjuvant elicited strong antibody responses, and to a lesser extend also cellular response (Qin et al. 2010). Both the PorA-Hoc and PorA-Soc (antigens of *Neisseria*) recombinant phages were highly immunogenic in mice and elicited strong antipeptide antibody titers even with a weak adjuvant such as Alhydrogel or no adjuvant at all. The data suggest that the phage T4 Hoc–Soc system is an effective system for display of peptides on an icosahedral capsid surface and may be as a powerful system for construction of the next generation of multicomponent vaccines (Jiang et al. 1997). Shivachandra et al. (2006) showed how to used T4 phage display system to develop customized multicomponent vaccines against anthrax. They hypothesized that multiple antigens fused to Hoc can be displayed on the same capsid, and such particles can elicit broad immunological responses. Anthrax toxin proteins: protective antigen (PA), lethal factor (LF), and edema factor (EF), and their functional domains were fused to Hoc with an N-terminal hexa-histidine tag, and the recombinant proteins were overexpressed in *E. coli* and purified. Using a defined in vitro assembly system, the anthrax-Hoc fusion proteins were efficiently displayed on T4 capsid, either individually or in combinations. Immunization of mice with T4 phage carrying PA, LF, and EF elicited strong antigen-specific antibodies against all antigens as well as lethal toxin neutralization titers. The triple antigen T4 phage elicited stronger PA-specific immune responses than the phage displaying PA alone. These features offer novel avenues to develop customized multicomponent vaccines against anthrax and other pathogenic diseases (Shivachandra et al. 2007). They also used Soc protein as a novel system for high-density display of multiple large anthrax toxins. Using a defined in vitro assembly system, anthrax toxins, protective antigen, lethal factor and their domains, fused to Soc, were efficiently displayed on the capsid. Both the N- and C-termini of the 80 amino acid Soc polypeptide can be simultaneously used to display antigens. T4-Soc platform is the one of the most robust phage display system reported to date (Li et al. 2007).

Further, the same group used T4 as a platform to construct experimental anti-HIV vaccines. The major capsid protein of HIV virion, p24-gag, was chosen as a model antigen to develop the system and determine the immunogenicity of the T4-displayed p24. Because of its high degree of sequence conservation among HIV isolates, p24 is an important target for HIV vaccine development (Frahm et al. 2004). Declining p24 antibody titers correlates with clinical deterioration and progression into AIDS (Sei et al. 1989). The T4-displayed p24 was highly immunogenic in mice, eliciting strong humoral and cellular immune responses. In addition, other HIV antigens, Nef and a novel gp41C-peptide trimer, were also targeted for display to assess the multivalent vaccine concept and the broad applicability of the system. The data show the efficient display of both single and multiple HIV antigens on the phage T4 capsid, thus offering foundations for designing novel HIV or other vaccines that have not been demonstrated by other vector systems (Sathaliyawala et al. 2006).

Ren et al. (2011) showed how to use T4 phage display system as an attractive approach for cancer therapy by employment of the Vascular Endothelial Growth Factor Receptor 2 (VEGFR2). In this experiment, mouse VEGFR2 was constructed on T4 phage nanometer particle surface as a recombinant vaccine. T4-mVEGFR2 recombinant vaccine was applied as an antitumor agent (Ren et al. 2011).

Whole phage particles can also be used to deliver vaccines by fusing immunogenic peptides to modified coat proteins (phage display vaccination), or by accepting a eukaryotic promoter-driven vaccine gene within the phage genome (phage DNA vaccination). Hybrid phage vaccination results from a combination of phage display and phage DNA vaccination strategies, showing the potential for evolution of phage vaccines. Phage vaccines could provide a key to unlock new approaches in combating bacterial and viral pathogens, and cancer diseases. The studies presented above provide abundant evidence that the phage T4 nanoparticle platform has the potential to enable human or veterinary vaccines (Qiu et al. 1999; Rao and Black 2010; Gao et al. 2010).

Vaccines are a cornerstone of modern public health and have been successfully used to control diseases. Despite this success, many current vaccines still have room for improvement, and some diseases require vaccine development. To change this need for further development in the vaccine field, Tao et al. (2013) developed an interesting bacteriophage T4-based technology that allows for concurrent delivery of desired DNA and proteins to mammalian cells. This technology allows for concurrent delivery of DNA and proteins, a possibility not available in the current standard approach. Phage T4-based technology could thus provide novel approaches for vaccination. Tao et al. (2013) generated phages that become unstable and release the genomic DNA back out of the head, leaving the empty head. Then, a DNA packaging motor was used to fill the empty head with their DNA of interest, with a capacity of up to ~170 kb. Additionally, to place a modified DNA into the phage head, proteins of interest could be added to decorate the head surface. Mammalian cells were treated with these phage-derived particles. Phage particles were taken up by mammalian cells. Proteins on the phage surface and DNA-encoded proteins were functional when delivered, and the proteins on the surface allowed targeted phage delivery to specified cells. Functional applicability of these engineered phage particles was further verified by immunization of mice. These results suggest that the technology warrants future investigation as a vaccine and/or therapeutic gene delivery platform. This potential vaccine platform offers important advantages including ease of preparation, large DNA carrying capacity, and ability to deliver therapeutic DNA and proteins (Kutzler and Weiner 2008; Hannigan and Grice 2013).

## Conclusions

Phage display is an exponentially growing research area. It usually applies filamentous phage strains: M13, fd, and f1. However, display systems based on bacteriophage T4 have also been developed, and they seem to be very promising. Bacteriophage T4 possesses unique features that make it a good tool for a multicomponent vaccine platform. Hoc/Soc-fused antigens can be assembled on the T4 capsid in vitro and in vivo. Using purified phage and functionally well-characterized antigens, the binding process can be tightly controlled in vitro, which increases the efficiency of T4 as a universal tool. The data show the efficient display of both single and multiple HIV antigens on the phage T4 capsid and offer insights for designing novel particulate HIV or other vaccines that have not been demonstrated by other vector systems. The T4 phage display system can also be used as an attractive approach for cancer therapy. T4-based phage display combined with affinity chromatography can be applied as a new method for bacteriophage purification.
